# Assessment of the BD MGIT TBc Identification Test for the Detection of *Mycobacterium tuberculosis* Complex in a Network of Mycobacteriology Laboratories

**DOI:** 10.1155/2014/398108

**Published:** 2014-01-23

**Authors:** Diana Machado, Jorge Ramos, Isabel Couto, Nureisha Cadir, Inácio Narciso, Elizabeth Coelho, Sofia Viegas, Miguel Viveiros

**Affiliations:** ^1^Grupo de Micobactérias, Unidade de Microbiologia Médica, Instituto de Higiene e Medicina Tropical, Universidade Nova de Lisboa (IHMT/UNL), Rua da Junqueira 100, 1349-008 Lisboa, Portugal; ^2^Centro de Recursos Microbiológicos (CREM), UNL, 2829-516 Caparica, Portugal; ^3^Instituto Nacional de Saúde, Ministério da Saúde de Moçambique, 264 Cidade de Maputo, Mozambique; ^4^Programa Nacional de Controlo da Tuberculose, Ministério da Saúde de Moçambique, 264 Cidade de Maputo, Mozambique; ^5^Centro de Malária e Outras Doenças Tropicais/LA, IHMT/UNL, Rua da Junqueira 100, 1349-008 Lisboa, Portugal

## Abstract

We evaluate the performance of the TBcID assay in a panel of 100 acid-fast bacilli cultures. Sixty-four isolates were TBcID positive for *Mycobacterium tuberculosis* complex (MTBC), whereas 36 gave negative results. These included 28 nontuberculous mycobacteria, one nonmycobacterial isolate, one *M. tuberculosis*, and six *M. bovis* BCG strains. This corresponds to a sensitivity of 90.14%, specificity of 100%, and positive and negative predictive values of 100% and 80.55%, respectively. The test is rapid, easy to perform and interpret, and does not require sample preparation or instrumentation. However, a negative result does not exclude the presence of a strain belonging to MTBC, especially when mutations in *mpb64* gene are present or some *M. bovis* BCG strains are isolated. The TBcID showed potential to assist in the identification of MTBC when the implementation and usage of molecular methods are often not possible, principally in resource-limited countries.

## 1. Introduction

The genus *Mycobacterium* comprises over 150 species of which more than 30 can cause disease in livestock, wildlife, and humans [1, 2]. Members of the *Mycobacterium tuberculosis* complex are the etiologic agents of tuberculosis and the responsible for about 8.6 million of new tuberculosis cases and 1.3 million deaths in 2012 [3]. Nontuberculous mycobacteria are a cause of opportunistic infections and are frequently encountered in clinical samples [1]. In some cases, clinical presentation of pulmonary disease caused by nontuberculous mycobacteria is very similar to that of tuberculosis [4]. Therefore, rapid diagnosis of patients with active tuberculosis is of major importance for the control of the disease demanding for reliable, easy to perform instrument-free identification assays, especially for low-income countries. Fast and accurate differentiation between *M. tuberculosis* complex and nontuberculous mycobacteria is essential, as it enables the implementation of appropriate measures to prevent the spread of the infection, allows the implementation of appropriate therapy, and prevents inappropriate drug susceptibility testing without species identification. Moreover, the differentiation of nontuberculous mycobacteria is important, since some of these species are resistant to most of the first line antibiotics used in tuberculosis therapy [4].

The conventional methods employed for the diagnosis of mycobacterial diseases rely on acid fast staining, culture, and phenotypic characterization. The development and implementation of liquid culture media allowed the reduction of the time for detection of positive cultures. From these, the BACTEC MGIT 960 is the most sensitive for recovery of mycobacteria from clinical samples [5]. After the positivity of a culture, the verification of the presence or absence of acid fast bacilli in the culture can be achieved by means of microscopy. Nevertheless, it does not distinguish between *M. tuberculosis* and nontuberculous mycobacteria. Usually, most laboratories in resource-limited settings use the labor intensive standard biochemical tests to identify *M. tuberculosis* complex, which requires subculture of mycobacteria on solid media and delay the result by several weeks. This process increases the turnaround time for reporting positive results. Nucleic acid probe and amplification based methods have been used for the identification of mycobacteria from cultures or directly from clinical samples thus reducing the time for diagnosis [6, 7]. However, since molecular methods do not distinguish between live and death bacteria, culture confirmation is mandatory. Moreover, these techniques require specific equipment, expensive reagents requiring refrigeration, and highly trained personnel.

Lateral flow assays, also called immunochromatographic assays, have been developed for the discrimination between *M. tuberculosis* complex and nontuberculous mycobacteria. These include the SD Bioline Ag MPT64 Rapid assay (Standard Diagnostics, Kyonggi-do, Korea), Capilia TB (TAUNS, Numazu, Japan), and the MGIT TBc Identification Test (Becton Dickinson Diagnostic Instrument Systems, Sparks, MD). These sandwich-type assays use a monoclonal antibody to detect the MPB64 protein (Rv1980c; also termed as MPT64), which is specifically secreted during growth by the *M. tuberculosis* complex [8]. The MPB64 is a 24 kDa protein, highly specific for the *M. tuberculosis* complex, except some variants of *Mycobacterium bovis* BCG [9, 10]. In this study we investigated the performance of the BD MGIT TBc identification test for the discrimination between *M. tuberculosis* complex and nontuberculous mycobacteria grown in both liquid and solid medium for the routine discrimination of *M. tuberculosis* complex in our setting and the reliability of the MPB64 protein for *M. tuberculosis* complex identification. This assay is intended to be used as a preliminary screening identification test when the hospital laboratories only perform acid-fast smear staining and culture and send their isolates to intermediate or reference laboratories for molecular identification at the species level and susceptibility testing of  *M. tuberculosis*.

This study was carried out as part of the training programs in TB laboratory diagnosis for Mozambique and other Portuguese speaking countries, created and implemented to assess the usefulness of this assays for routine identification of *M. tuberculosis* in the network of hospital mycobacteriology laboratories in these countries as well as in Portugal.

## 2. Methods

### 2.1. Mycobacterial Strains and Culture

A total of 100 culture isolates received from seven hospitals of the Lisbon Health Region during the training period of three months were included in this study. These comprised 71 strains of the *M. tuberculosis* complex, 28 nontuberculous mycobacteria, and one acid-fast bacilli other than mycobacteria (Table 1). *M. tuberculosis* H37Rv and *M. bovis* BCG Pasteur strain were used as controls. As an intermediate level laboratory in the network of hospital mycobacteriology laboratories in Lisbon, the Mycobacteriology Laboratory of the IHMT/UNL only receives acid-fast smear positive cultures for molecular identification and drug susceptibility testing from the hospital laboratories. All isolates were processed for Ziehl-Neelsen staining and inoculated into MGIT culture tubes and Lowenstein-Jensen slants. Samples were digested and decontaminated, by the standard sodium hydroxide-N-acetyl-L-cysteine (NaLC-NaOH) method [11]. Once a positive signal was given by the BACTEC system, a Ziehl-Neelsen staining was performed and the presence or absence of serpentine cording morphology was observed. Only acid-fast bacilli positive cultures were included in the study. Presence of contamination was evaluated by inoculation of the cultures into blood agar plates and Ziehl-Neelsen staining.

### 2.2. TBcID Assay

The TBcID assay (Becton Dickinson Diagnostic Instrument Systems, Sparks, MD) consists of a nitrocellulose membrane on a test device with immobilized anti-MPB64 mouse monoclonal antibodies conjugated with gold colloidal for the detection of the MPB64 protein. In the presence of a sample, the antibody-colloidal gold conjugate binds to the MPB64 antigen and flows laterally through the membrane until the reaction zone. Here, the complex will be captured by a second antibody specific for MPB64. If the MPB64 protein is present, a purple band will be developed. Each TBcID device was inoculated with 100 *μ*L of a positive MGIT culture. All cultures were tested with growth units above 100 (GU ≥ 100) and between day 1 and day 5 after positivity within the MGIT system. Selected *M. tuberculosis* isolates from the same collection were evaluated with cultures grown in Lowenstein-Jensen. For that, one loopful of colonies was suspended in 200 *μ*L of extraction buffer (phosphate buffer with 0.05% Tween 20 and 0.02% sodium azide) and 100 *μ*L of the suspension used in the assay. The results were interpreted 15 min after application of the sample. A positive result was indicated by the development of two purple bands, one in the control zone (C) and another in the test zone (T). The presence of the control band alone indicates a negative result.

### 2.3. Confirmatory Identification Tests

All the results obtained with TBcID were compared with Accuprobe MTBC culture identification test (GenProbe Inc., San Diego, CA) as the “gold standard” for the identification of the* M. tuberculosis* complex. Briefly, 1 mL of the each culture was centrifuged during 10 min at 13000 rpm and the pellet was used for hybridization according to the instructions of the manufacturer. The isolates that were negative for *M. tuberculosis* complex by *Accu*probe were identified using the Genotype CM/AS (Hain, Nehren, Germany) according to manufacturer's instructions. Total genomic DNA for the Genotype CM/AS assays was extracted from the cultures using the QIAamp DNA mini kit (QIAGEN, GmbH, Hilden, Germany) according to the manufacturer's instructions.

### 2.4. Assessment of Discordant Results

The isolates that were positive for *M. tuberculosis* complex with *Accu*probe but negative with the TBcID were tested using the Genotype MTBC (Hain) assay, according to manufacturer's instructions. Mutations in the *mpb64* gene of *M. tuberculosis* were analyzed by PCR amplification and DNA sequencing using the primers *mpb64*-F30 and *mpb64*-R433, *mpb64*-F404 and *mpb64*-R891, described elsewhere [12]. The reaction mixtures were prepared for a total reaction volume of 50 *μ*L consisting of 1x Taq buffer (Fermentas, Ontario, Canada), 1.5 mM MgCl_2_, 200 mM of each dNTP, 10 pmol of each primer, 1.5 U Taq DNA Polymerase (Fermentas), and 5 *μ*L of chromosomal DNA. The PCR reactions were performed with the following amplification profile: initial denaturation at 94°C for 5 min, followed by denaturation at 94°C for 1 min, annealing at 62°C for 1 min, and extension at 72°C for 1 min during 40 cycles. The final extension occurred at 72°C for 10 min. PCR products were sequenced with an ABI Prism 3130 capillary sequencer (Applied Biosystems, Foster City, CA) and the BigDye terminator kit (ABI Prism).

### 2.5. Performance Analysis

The sensitivity, specificity, and positive and negative predictive values of the TBcID assay were determined using the results of the Accuprobe MTBC culture identification test as the gold standard.

## 3. Results 

The results obtained are summarized in Tables 1 and 2. Of the 100 mycobacterial cultures tested, 64 were correctly identified as *M. tuberculosis* complex by the TBcID. The assay failed to detect one *M. tuberculosis* isolate that carried a mutation in the *mpt64* gene and all *M. bovis* BCG tested (*n* = 6). We did not observe cross-reaction with any of the 28 nontuberculous mycobacteria and the one nonmycobacterial acid-fast bacilli culture tested. These correspond, in this study, to a sensitivity of 90.14%, specificity of 100%, positive predictive value of 100%, and negative predictive value of 80.55% of the TBcID assay for the identification of the *M. tuberculosis* complex.

From the seven false negatives yielded by TBcID one corresponded to an *M. tuberculosis *strain as determined by Accuprobe. To evaluate if the negative result was due to a reduced amount of secreted MPB64 protein necessary for the detection, this isolate was subcultured and the test was repeated. The result was again negative. After this, the entire *mpb64* gene was sequenced and an insertion of two bases (CG) at position 335 of the gene was detected. This frame shift resulted in the generation of a premature stop codon at amino acid position 167, truncating the protein. The remaining six isolates were identified as *M. bovis* BCG by the Genotype MTBC assay and these isolates were later on found to belong to one child BCGitis and from patients that are being monitored for bladder cancer immunotherapy [13]. Testing by polymerase chain reaction with the four primers confirmed that the *mpb64* gene is absent in these strains.

**Figure 1:**
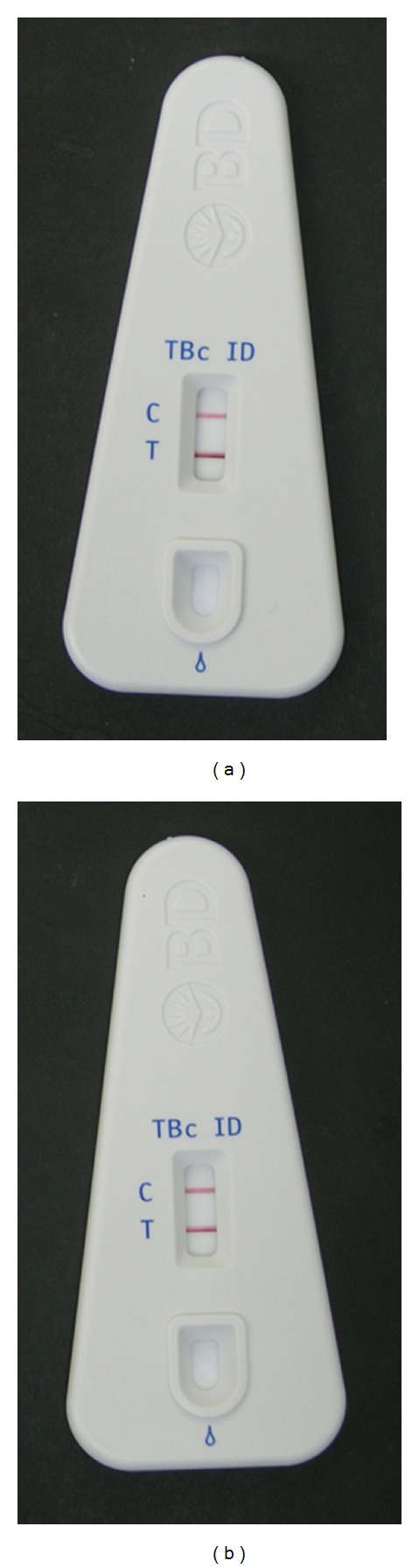
Identification of *M. tuberculosis* complex isolates by the MGIT TBc ID assay from (a) positive MGIT culture and (b) Lowenstein-Jensen slants. In the Figure it is shown the result of the TBcID for the same isolate when grown in different culture media. The positive result is indicated by the development of two purple bands, one in the control zone (C) and another in the test zone (T).

The manufacturer's instructions of the TBcID system recommend its use with isolates grown in liquid media only. Nevertheless, we decided to test the capability of the test to detect MPB64 protein in strains grown on solid media. For that we selected 25 *M. tuberculosis* isolates from the panel of isolates grown in liquid media previously evaluated. The test demonstrates a good performance for detection of *M. tuberculosis* complex from solid cultures as all isolates were correctly identified as *M. tuberculosis* complex (Figure 1 and Table 3).

## 4. Discussion 

In this work, the performance of the BD MGIT TBc identification test was evaluated for the identification of *M. tuberculosis* complex and differentiation from nontuberculous mycobacteria. We did not detect false positive results. However, the TBcID assay yielded seven false negative results. The assay demonstrates 100% of specificity which is similar to that published in other studies [14–16]. Nevertheless, the sensitivity was found to be 90.14%, which is lower than that reported by other authors (between 95.2 and 100%) [14–16]. This fact can be explained by the false negative results obtained.

The occurrence of false negatives can be due to the absence of the MPB64 protein or mutations in the coding gene, *mpb64*. Several studies have reported false negatives results for several BCG strains [14, 17, 18]. Among the *M. bovis* BCG variants, some of them do not produce the MPB64 antigen, whereas others are good secretors of this antigen [19]. This difference is due to the deletion of the *mpb64* gene together with the RD2 [20]. One of the strains included in this study was the BCG Pasteur, already described as a nonproducer of the MPB64 antigen [21, 22]. In Portugal, BCG SSI (strain 1331) is used for vaccination and BCG Medac is used for treatment of noninvasive urothelial bladder carcinoma and thus we assume that we have found both strains in our study, since one strain came from a child who developed osteomyelitis after BCG vaccination and five strains were isolated from patients undergoing cancer therapy [13]. Another possible explanation for the occurrence of negative results is the presence of mutations within the *mpb64* gene. Several mutations are reported in the literature for this gene [12, 15, 16, 18, 22–24]. In this study, we detected a GC insertion at nt 335, which resulted in a truncated protein and a TBcID negative result for this *M. tuberculosis* strain. As far as we know, this mutation has not been previously reported. Further, due to the existence of some strains of *M. tuberculosis* with delayed MPB64 secretion some cultures might lead to erroneous reporting of negative results. Vadwai et al. [25] propose that a culture must be tested with GU ≥ 300 to avoid false negative results. In our study, we did not detect false negative results using as cutoff MGIT cultures with GU ≥ 100.

Differentiating *M. tuberculosis* complex from nontuberculous mycobacteria as soon as possible is important, mainly in situations in which nontuberculous mycobacteria strains represent a considerable portion of mycobacteria isolated [26]. The nontuberculous mycobacteria and one acid fast bacilli other than mycobacteria tested in this study correctly provided true negative results. Serpentine cord morphology can be used for rapid presumptive identification of *M. tuberculosis* in liquid culture and as a guide for the selection of auxiliary tests. Using this feature, we were able to improve the sensitivity of the test since the combination of the serpentine cording morphology with the TBcID comparing with AccuProbe assay as the gold standard corresponds to a sensitivity and specificity of 100% and identical positive and negative predictive values (data not shown). Similar results were reported by others [27]. The capacity of the TBcID was also evaluated for the identification of a subset of 25 *M. tuberculosis* strains grown on solid media, and all the 25 isolates were correctly identified as *M. tuberculosis* complex.

The main advantages of the test are the cost and rapidity. However, it has some disadvantages. The method (i) cannot be applied directly to clinical samples; (ii) does not allow the identification at species level; and (iii) requires further confirmatory tests for identification at species level. The MPB64 protein is the target of the test but it is also its weak point. All negative results suspected for *M. tuberculosis* complex had to be tested with other methodologies. Noteworthy, the isolation of *M. bovis* from human samples is uncommon, but sporadically, BCG strains are isolated from patients receiving BCG immunotherapy. The fact that all of our BCG strains are negative for MPTB64 can be useful to assist in the detection of BCG strains since it can be used as a screening tool in combination with molecular methods. Our results show that the TBcID is not an alternative to the Accuprobe system, at least in our work algorithm, due to the false negative results presented and this also precludes its use for confirmatory laboratory diagnosis of tuberculosis infections. Another limitation is related to the safety measures. The test necessitates biosafety level 3 conditions to be applied as it involves a considerable bacterial inoculum. When these conditions are not present, the test must be performed with heat-inactivated cultures [14].

The BD MGIT TBc identification test showed potential to assist in the identification of *M. tuberculosis* complex and differentiation from nontuberculous mycobacteria when the implementation and usage of molecular methods are often not possible, principally in resource-limited countries. The test is simple, rapid, easy to perform and interpret, and does not require sample preparation or instrumentation. In Mozambique, in 2012 the TB Reference Laboratories from Maputo and Beira have introduced the immunochromatographic assay, a very important step in a country for which very limited information regarding the occurrence of nontuberculous mycobacteria is available [28]. In 2012, among all positive cultures, 20.5% were from single or mixed infection from nontuberculous mycobacteria, a strong evidence of the high prevalence of nontuberculous mycobacteria in this country that are often misidentified and might be considered as multidrug resistant tuberculosis.

This study demonstrated the usefulness of the immunochromatographic assays for routine identification of *M. tuberculosis* in a network of mycobacteriology laboratories as preliminary screening identification test of cultures to be sent to the intermediate or reference laboratory as part of the network of TB laboratories of the national tuberculosis control programs.

## Figures and Tables

**Table 1:** Identification of *M. tuberculosis* complex and differentiation from nontuberculous mycobacteria by the TBcID.

Mycobacterial species (*n*)	TBcID	
	Positive	Negative
*M. tuberculosis *complex (71)	64	7
Nontuberculous mycobacteria (28)	0	28
AFB high G+C bacteria (1)	0	1

AFB: acid-fast bacilli.

**Table 2:** Comparison of the results obtained by the TBcID and the Accuprobe assay.

Mycobacterial species (*n*)	TBcID		Accuprobe	
	Positive (*n*)	Negative (*n*)	Positive (*n*)	Negative (*n*)
*M. tuberculosis *complex				
*M. tuberculosis *(64)	63	1	64	0
*M. bovis *BCG (6)	0	6	6	0
*M. africanum *(1)	1	0	1	0
Nontuberculous mycobacteria				
*M. abscessus *(2)	0	2	0	2
*M. avium *(4)	0	4	0	4
*M. intracellulare* (5)	0	5	0	5
*M. chelonae *(2)	0	2	0	2
*M. fortuitum *(1)	0	1	0	1
*M. genavense *(2)	0	2	0	2
*M. gordonae *(4)	0	4	0	4
*M. kansasii *(1)	0	1	0	1
*M. marinum *(2)	0	2	0	2
*M. peregrinum *(1)	0	1	0	1
*M. scrofulaceum *(1)	0	1	0	1
*M. szulgai *(1)	0	1	0	1
*M. xenopi *(1)	0	1	0	1
*M. ulcerans *(1)	0	1	0	1
Nonmycobacteria				
AFB high G+C bacteria (1)	0	1	0	1

BCG: Bacillus Calmette-Guérin. AFB: acid-fast bacilli.

**Table 3:** Results of the TBcID for a subset of 25 *M. tuberculosis* complex strains using different culture media.

Strain ID	TBcID result	
	MGIT	Lowenstein-Jensen
22	(+)	(+)
28	(+)	(+)
56	(+)	(+)
57	(+)	(+)
58	(+)	(+)
61	(+)	(+)
70	(+)	(+)
74	(+)	(+)
75	(+)	(+)
76	(+)	(+)
77	(+)	(+)
78	(+)	(+)
79	(+)	(+)
80	(+)	(+)
81	(+)	(+)
87	(+)	(+)
90	(+)	(+)
91	(+)	(+)
94	(+)	(+)
95	(+)	(+)
96	(+)	(+)
97	(+)	(+)
98	(+)	(+)
99	(+)	(+)
100	(+)	(+)

Total (25)	25	25

(+) positive result.
